# Neglected Fournier's Gangrene Caused by *Acinetobacter baumannii*: A Rare Case Report

**DOI:** 10.1155/2016/8461354

**Published:** 2016-09-20

**Authors:** Arif Emre, Mehmet Sertkaya, Sami Akbulut, Yakup Duman, Ilhami Taner Kale

**Affiliations:** ^1^Department of Surgery, Kahramanmaras Sutcu Imam University Faculty of Medicine, 46100 Kahramanmaras, Turkey; ^2^Department of Surgery and Liver Transplant Institute, Inonu University Faculty of Medicine, 44280 Malatya, Turkey; ^3^Department of Plastic and Reconstructive Surgery, Kahramanmaras Sutcu Imam University Faculty of Medicine, 46100 Kahramanmaras, Turkey

## Abstract

Fournier's gangrene, rare but life threatening disease, is characterized by an acute necrotic infection of the scrotum, penis, or perineum. Fournier's gangrene is a mixed infection caused by both aerobic and anaerobic bacteria. Fournier's gangrene caused by multidrug resistant* Acinetobacter baumannii* have been reported rarely. The mainstay of treatment is prompt recognition and a combination of antibiotics with radical debridement. We describe a case of a 56-year-old male patient presenting with neglected Fournier's gangrene caused by* Acinetobacter baumannii*. Many treatment modalities including broad-spectrum antibiotics, aggressive debridement, negative pressure wound therapy, diversion colostomy, and partial-thickness skin grafts were applied to save the patient's life.

## 1. Introduction

Fournier's gangrene is a necrotizing infection of the perineum and genitalia comprising the scrotal and perianal regions skin, subcutaneous tissue, and fascia. This serious disease is characterized by rapidly progressive inflammation and necrosis [1]. This disease which is frequently misdiagnosed as cellulitis is initiated by local trauma in patients with predisposing factors like diabetes, malnutrition, and immunosuppression. Many aerobic and anaerobic microbial agents including* Acinetobacter baumannii* are involved in its etiology [2]. Diagnosis of the disease should be placed without delay and treatment should be initiated immediately, because it shows very fast progression. Aggressive debridement should be implemented quickly to limit the spread of infection when necessary. Otherwise, it can cause severe morbidity and mortality. Herein, we present a case of neglected Fournier's gangrene caused by multidrug resistant* Acinetobacter baumannii*.

## 2. Case Report

**Figure 1:**
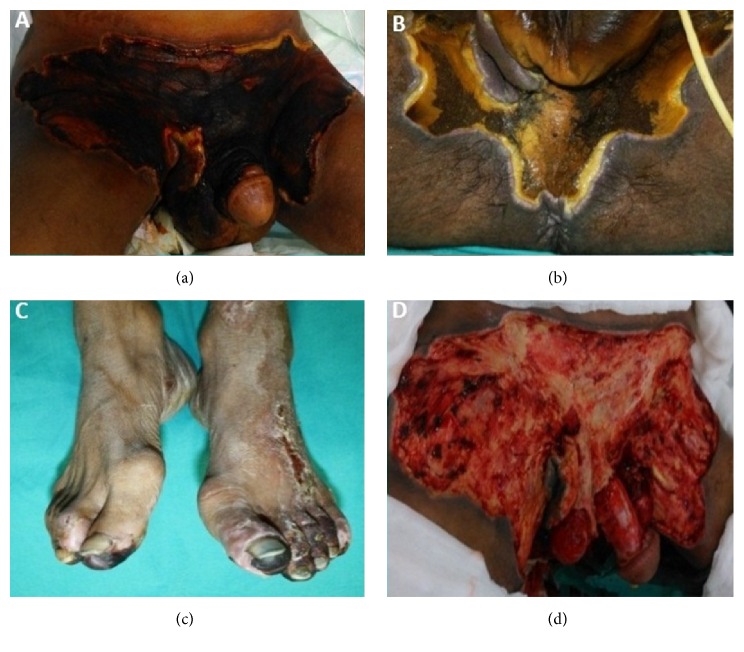
Fournier's gangrene caused by* Acinetobacter baumannii*. Anterior aspect of the patient's pelvic wound before the surgical debridement (a), posterior aspect of the patient's pelvic wound before the surgical debridement (b), the patient's foot wound before the surgical debridement (c), and the patient's wound after the surgical debridement (d).

**Figure 2:**
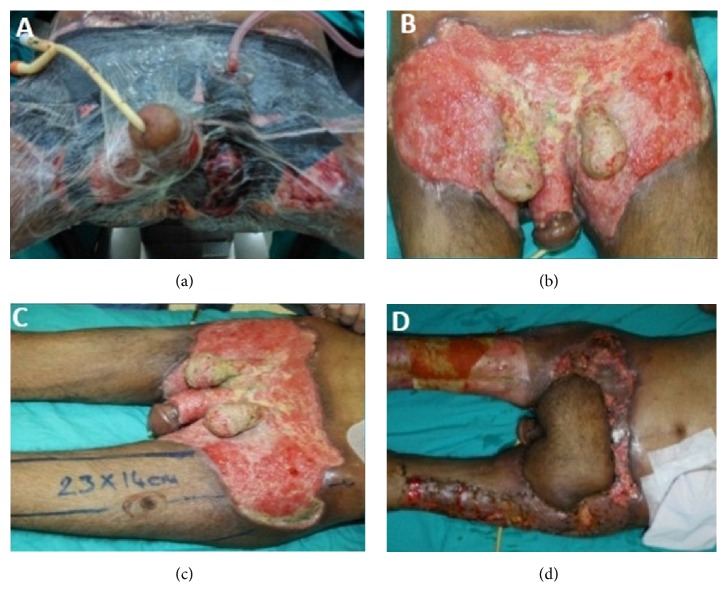
Wound dressing, vacuum-assisted wound closure (a), the patient's wound after forty-five days of the surgical debridement (b), and partial-thickness skin grafts taken from the front of the thigh and also anterolateral thigh flap with a proximal pedicle (c, d).

A 56-year-old man with Fournier's gangrene was referred from another medical center to our clinic due to worsening symptoms. He worked as a farmer and was from a low economic and sociocultural situation. He did not have any significant disease except for idiopathic cachexia. He had a history of scrotal abscess associated with urinary tract infection for forty days. In spite of the medical treatment period of forty days, expected success in its treatment could not be provided. When he was admitted to our department, he had neither fever nor other septic conditions. An initial physical examination demonstrated that he was cachectic and his body mass index was about 15 kg/m^2^. Oral and parenteral nutritional support began to improve the patient's immunological and nutritional parameters. It was noticed that although the great majority of gangrene was dry, very small part of wound was wet gangrene especially the edges during examination of the wound (Figures 1(a)-1(b)). He also had partial gangrene in both toes (Figure 1(c)). Cultures of samples taken from the perineal wound site grew* Acinetobacter baumannii* susceptible to colistimethate sodium (colistin). The toes wound culture also grew* Trichosporon asahii* susceptible to fluconazole. On the 5th day of treatment, he underwent debridement of extensive necrotic tissue at the edge of sufficient blood supply to the wound (Figure 1(d)). Additionally, colostomy was opened to protect the wound. Negative pressure wound therapy was applied to the surface of the debrided area and intermittently continued for 45 days (Figure 2(a)). The wound was convenient to dressing with flapping in the forty-five days after the surgical debridement (Figure 2(b)). Defect was closed with partial-thickness skin grafts taken from the front of the thigh and also anterolateral thigh flap with a proximal pedicle 50 days after the first application (Figures 2(c)-2(d)). He was discharged from the hospital 80 days after the first admittance.

## 3. Discussion

Fournier's gangrene was first described in 1883 by the French venereologist Jean Alfred Fournier as a disease of young adults of unknown cause and sudden onset of pain and swelling and rapid progression to gangrene in the skin of the penis and scrotum [1]. Although etiology of the disease was unclear in the past, recent studies pointed out that its etiology is generally a pathological process from the underlying skin, urinary tract, or colorectal area infections [2]. Contributing factors to worsening of the disease are poor self-care, malnutrition, systemic immunosuppression, diabetes mellitus, arterial hypertension, chronic renal failure, systemic disorders, malignant neoplasms, local trauma, chronic alcoholism, and steroids therapy [1, 3]. This present case had idiopathic cachexia, poor self-care, and urinary tract infection.

The most frequently found pathogen is polymicrobial organism (54%), followed by* Escherichia coli* (46.6%) and* Streptococcus* (36.8%). Less frequently encountered pathogens also include* Bacteroides*,* Enterobacter*,* Staphylococcus*,* Enterococcus*,* Pseudomonas*,* Corynebacterium*,* Klebsiella pneumoniae*, and* Acinetobacter baumannii* [4, 5].* Acinetobacter* spp. are a group of Gram-negative bacteria belonging to the family Moraxellaceae and are important soil organism and able to survive on various surfaces (both moist and dry) in the hospital environment, thereby being an important source of infection in debilitated patients. These bacteria are innately resistant to many classes of antibiotics [5, 6]. In this case,* Acinetobacter baumannii* obtained from samples taken from the wound was multidrug resistant except for colistin.

When it is suspected that Fournier's gangrene develops, it should be treated urgently with empirical broad spectrum intravenous antibiotic therapy and early aggressive surgical debridement and the liquid electrolytes replacement and when blood transfusion was required, hemodynamic stabilization should be provided by applying repeated transfusions [2, 3]. Although the patient was started on the antibiotics on admission, we believe that it was late for surgical debridement. When we accepted the patient in our center, gangrene had spread over a wide area. As a result of the cultures, inappropriate antibiotics which were empirically given at the beginning of treatment can be replaced with appropriate ones. The aim of applied surgery is that all necrotic tissues must be excised until the limit of sufficient tissue perfusion is reached and then adequate hemostasis should be provided. The removed tissue should be sent for frozen section examination for the definitive histologic diagnosis and the agents causing the disease should be determined with the help of Gram stain and culture examinations. If necessary, those repeated debridement procedures should not be avoided.

Supportive care should be provided by respiratory support, cardiovascular monitoring, required hemodialysis and blood transfusions, and the treatment with oral or IV hyperalimentation or both at the same time to include nutritional therapy. Low-dose heparin may be given to the patient to prevent deep vein thrombosis. Recent studies have shown that hyperbaric oxygen therapy has been demonstrated to reduce both preoperative and postoperative morbidity and mortality and contribute to healing the wounds [7]. Also it has been shown in the patients who have Fournier gangrene that the negative pressure vacuum applications have a positive impact on wound healing and are well tolerated by patients, before covering with skin grafts and after grafting [8, 9].

Unwanted functional and aesthetic problems including scrotal, penile, and perianal tissue defects after treatment of Fournier's gangrene are a serious matter. These defects are usually reconstructed with tension-free closure, skin grafts, or flaps. Although a consensus about it is not yet formed, if it is possible, tension-free closure is preferable for good tissue healing and skin color harmony. In the patients who grow tension as a result of the primary closure defects, especially patients with large defects, flaps or grafts should be used. Karian et al. recommend primary closure, anticipating that the tension does not develop in the case when scrotal defects are limited to the mouth of the scrotum and are less than 50% of scrotal area. Otherwise, that is, in the case in which tension occurs, they suggest application of local advancement flap or leaving secondary healing. If scrotal defects are greater than 50% and are stretched out of the scrotum, they suggest application of split-thickness skin graft +/− tissue adhesive agents or flap reconstruction +/− tissue adhesive agents [10]. The most common complications of skin grafts are bleeding, graft contraction, and loss of graft caused by the infection. The flap reconstructions provide durable protection to the testicles. Additionally they have less contracture rates.

As a result, Fournier's gangrene has the ability to make rapid progress and day by day leads to injury in more and more areas. When the time of intervention is delayed, its treatment becomes complicated. Both of support treatment and the reconstruction of defects are difficult with delaying time. Just as in our case of neglected Fournier's gangrene caused by* Acinetobacter baumannii*, early surgical debridement is the first requirement for the desired good results [11].

Despite the wide spectrum of antibiotics used today and improvements in surgical techniques, Fournier's gangrene still leads to serious morbidity and mortality. Fournier's gangrene in especially neglected cases who are surgically delayed is rapidly progressive and spreads over large areas. It has been encountered in the undesirable aesthetic results in the case of large tissue defects from wide surgical debridement.
